# Trust in the Police during the Pro-Democracy Movement in Hong Kong: Psychosocial Factors of Perceived Procedural and Distributive Justice

**DOI:** 10.3390/ijerph19116495

**Published:** 2022-05-26

**Authors:** Heng Choon (Oliver) Chan

**Affiliations:** Teaching Laboratory for Forensics and Criminology, Department of Social and Behavioral Sciences, City University of Hong Kong, Hong Kong SAR, China; oliverchan.ss@cityu.edu.hk; Tel.: +852-3442-9223

**Keywords:** police procedural justice, police distributive justice, perceptions, psychosocial factors, anti-extradition movement, Hong Kong

## Abstract

Hong Kong has experienced social unrest in response to the proposed anti-extradition bill since early June 2019. Demonstrations and rallies have often ended in violent clashes between protestors and the police. Based on a sample of 1024 Hong Kong adults, this study explored the psychosocial factors underlying public perceptions of police procedural and distributive justice among Hong Kongers. Testing the propositions of several criminological theories (i.e., neutralization theory, the general aggression model, general strain theory, and self-control theory), the findings indicated that men reported significantly more positive general perceptions of police procedural and distributive justice, better general mental health, and more negative attitudes toward violence than women did. Young adults perceived significantly higher levels of police general, procedural, and distributive justice than did their middle-aged and older counterparts, who reported significantly better general mental health and greater self-control. Multivariate analyses indicated that across all age groups, better general mental health, greater self-control, and more negative attitudes toward violence were significantly associated with positive perceptions of police general, procedural, and distributive justice. This study concludes with practical guidance for enhancing public perceptions of police procedural and distributive fairness.

## 1. Introduction

Since early June 2019, Hong Kong has experienced mass demonstrations that have ranged from peaceful rallies to violent clashes between protestors and the police. The social unrest was originally focused on a bill that would have made it easier to extradite Hong Kongers to mainland China. This bill, the Fugitive Offenders and Mutual Legal Assistance in Criminal Matter Legislation (Amendment) Bill 2019 (the Anti-Extradition Law Amendment Bill (ELAB)), was proposed by the Hong Kong government in February 2019. The bill was proposed to amend the Fugitive Offenders Ordinance (Cap. 503) with respect to special surrender arrangements not previously covered by legislation, and to amend the Mutual Legal Assistance in Criminal Matters Ordinance (Cap. 525) by permitting mutual legal assistance arrangements between Hong Kong and other jurisdictions, including the PRC [1]. It was sparked by the murder of a young woman by her boyfriend, a young man from Hong Kong, in Taiwan. Pervasive public criticism of the bill culminated in a series of Anti-ELAB social protests, such as large-scale demonstrations, rallies, sit-ins, and general strikes, throughout Hong Kong. The social unrest occurred for nearly seven months, until late January 2020, when the COVID-19 pandemic began to affect the routines of Hong Kongers [2]. Even during the early stages of the pandemic, in the first half of 2020, small-scale (often illegal, as the police denied the applications to hold rallies) rallies and protests (some held on weekends or during weekday lunchtimes) continued to occur sporadically. These demonstrations regularly ended in violent clashes between a small number of protestors and the police; the latter received the greatest criticism for their excessive use of force. This social chaos was exacerbated by the enactment of the Hong Kong National Security Law, which took effect on 1 July 2020, marking the 23rd anniversary of Hong Kong’s return to the People’s Republic of China (PRC). The law was enacted primarily to: (1) ensure the resolute, full, and faithful implementation of the policy of One Country, Two Systems; (2) safeguard national security; (3) prevent, suppress, and impose punishment for the offenses of secession, subversion, organization, and perpetration of terrorist activities and collusion with a foreign country or other external elements to endanger national security; (4) maintain the prosperity and stability of Hong Kong; and (5) protect the lawful rights and interests of Hong Kong residents [1]. Since its enactment, public anger has been directed not only at both the Hong Kong and the mainland Chinese governments, but also at the police. Public outrage against the police has largely focused on officers’ excessive use of force and unjust treatment of citizens.

“Legitimacy”, in a broad sense, is a property of an authority or institution that leads people to perceive that authority or institution as entitled to deference and obedience [3,4]. Accordingly, policing agencies that effectively deter crime, arrest offenders, and fairly distribute services across the community are traditionally seen as legitimate [5]. Tyler [6] postulated that people’s perceptions of police legitimacy are influenced mainly by whether they perceive the police to be following fair and equitable procedures (i.e., procedural justice or fairness) in both their decision making and their behavior during police–citizen interactions. Perceptions of legitimacy are partly impacted by the perceived fairness of the distribution of police services (i.e., distributive justice or fairness). Put simply, procedural justice is concerned with the means of decision making (i.e., predictors), while distributive justice is concerned with the ends of decision making (i.e., outcomes) [7]. Both perceived procedural justice and distributive fairness promote overall police legitimacy, which in turn improves legal compliance and cooperation with the police.

Over the years, studies on public perceptions of police procedural and distributive justice have been extensively studied (see, e.g., [8]). Recent studies in this area have drawn samples from various populations, including arrestees [9], female offenders and inmates [10,11], victims of crime [12], and nonoffenders and nonvictims (e.g., vehicular drivers by Antrobus et al. [13]; immigrants by Pryce et al. [14]). Research has obtained mixed findings regarding sex-based differences in perceptions of procedural and distributive justice in criminal justice contexts. For instance, Kulik and colleagues [15] failed to find any differences between male and female participants in either their treatment by police or their judgments of distributive and procedural justice in a judicial context. Conversely, Felson and Pare [16] found that female victims of sexual and physical assault were significantly more likely than male victims to report dissatisfaction with the police, as they viewed themselves as discriminated against in the criminal justice system. Antrobus and colleagues [13] found that males (vs. females) and those who were more educated were less likely to report an obligation to obey the police. Concerning the effect of age, most studies have found that older individuals are more likely to have positive perceptions of police procedural and distributive justice, making them more inclined to obey the police; e.g., [17,18]. However, Tankebe [17] reported that older, less educated Ghanaian individuals were less likely to trust the police, making them more willing to support violent self-help mechanisms such as vigilantism, particularly when security provision by the police was regarded as ineffective.

Despite the plethora of literature on the topic, little is known about public perceptions of police procedural and distributive justice in Chinese contexts, particularly against the background of a massive social movement conducted over an extended period (e.g., the Anti-ELAB Movement). A rare empirical study on this topic that sampled participants from mainland China was conducted by Sun and colleagues [18], who sought to determine whether Tyler’s Western model [6] could be applied in a Chinese context. Similar to the Western findings, procedural justice was found to significantly predict perceptions of police legitimacy and willingness to cooperate with the police in mainland China. Police effectiveness was the strongest predictor of perceptions of police legitimacy, through which both distributive justice and effectiveness had an indirect impact on the willingness to cooperate [18]. A more recent study by Liu and Liu [19] examined the role of police legitimacy in predicting compliance with the law in a sample of mainland Chinese youth. The participants’ perceptions of procedural justice and shared values were strongly associated with their support for the police, which in turn fostered their compliance with the law. Perceived distributive fairness was also found to exert an independent effect on compliance: for those who had been questioned by the police, perceived distributive fairness was negatively associated with compliance with the law.

A study on the Anti-ELAB Movement in Hong Kong was conducted by Stott and colleagues [20] to explore the social psychological dynamics throughout this social movement by utilizing the Elaborated Social Identity Model of crowd behavior. They observed three key phases of evolution in the patterns of collective action and differences in policing approaches. The early days of the social movement were focused primarily on preventing the implementation of the controversial legislation (i.e., ELAB), but soon spread and changed in form as a function of the use of crowd-control tactics by the police. The police inaction in some critical incidents (e.g., the Yuen Long attack on 21 July 2019 and the Prince Edward MTR station incident on 31 August 2019) helped amplified perceptions of police illegitimacy that subsequently radicalized protestors [2]. The Yuen Long attack on the evening of 21 July 2019, also known as the “721 incident”, refers to a mob attack that occurred in Yuen Long, a town in the New Territories. An armed mob of suspected triad members dressed in white indiscriminately attacked civilians in the MTR station and nearby streets with steel rods and rattan canes. It was claimed that despite over 24,000 calls to the 999-emergency hotline, the police only arrived 39 minutes after the attacks and 1 minute after the mob had left the Yuen Long MTR station. The Prince Edward MTR station incident on the night of 31 August 2019, also known as the “831 incident”, refers to an incident in which the police were alleged to have indiscriminately attacked passengers while arresting protestors who were returning home through the Prince Edward station. Maguire [21] commented that the coercive policing practices adopted by the Hong Kong police during the latter part of the social movement harmed their relationships with the public, and in turn diminished the perceived legitimacy of the police and undermined human rights. In his recent study on the Anti-ELAB Movement, Ho [22] contented that the shift to paramilitary policing approach in protest management in recent years has in turn led to a further decline in public perceptions of police legitimacy across the Hong Kong population.

Given the scarcity of studies assessing Chinese participants’ perceptions of police procedural and distributive justice, as well as studies examining the validity of Tyler’s work in a Chinese context, this study investigated public perceptions of police procedural and distributive justice in another major Chinese society, Hong Kong. It explored the psychosocial factors that have influenced perceptions of police procedural and distributive justice among Hong Kongers during the recent social turmoil by testing several mainstream criminological theories (examining attitudes toward violence based on neutralization theory and the general aggression model; examining general mental health based on general strain theory; and examining self-control based on self-control theory). The primary aim of this study was to capture how Hong Kongers perceived police procedural and distributive justice within a specific timeframe (i.e., during and immediately after the 2019–2020 Anti-ELAB Movement).

Against this background, this study primarily aimed to explore the theoretical perspective of Hong Kongers’ perceptions of police procedural and distributive justice. Three psychosocial factors were examined, namely neutralization theory and the general aggression model (the participants’ attitudes toward violence; cognitive aspect), general strain theory (the participants’ affects and mental health; affective aspect), and self-control theory (the participants’ impulsivity; personality aspect). These theoretical models were used to examine the different psychosocial features of Hong Kongers’ perceptions of police procedural and distributive justice. In addition to advancing knowledge, this study may offer important practical guidance for promoting positive police–citizen engagement and public trust in the police. Timely and effective interventions are essential to prevent the further deterioration of police–citizen relations in Hong Kong, as witnessed during the massive social movements that have occurred in recent years (e.g., the 2014 Umbrella (or Occupy) Movement, the 2016 Fishball Revolution (or Mongkok Civil Unrest), and the 2019–2020 Anti-ELAB Movement). Hong Kongers’ perceptions of criminal justice authorities, such as the police, may affect their perceived obligation to obey the law.

## 2. Theoretical Background

Criminal attitudes are regarded as one of the “big four” risk/needs factors key to predicting and managing the risk of reoffending [23]. The literature has consistently found that habitually violent individuals tend to hold more favorable attitudes toward violence [24,25,26,27]. For example, Hopkins [28] asserted that individuals who hold adversarial attitudes toward specific groups of individuals (e.g., racist, homophobic, or Islamophobic attitudes) have a heightened tendency to perpetrate violence against these groups. Other studies have also demonstrated positive relationships between pro-violence attitudes and the subsequent perpetration of actual violence, especially among children and adolescents [29,30]. Broadly defined as crime-supportive cognitions, pro-criminal or pro-violence attitudes consist of a wide range of thoughts and beliefs [31].

Neutralization theory has often been used to describe the origins and functions of many cognitions supportive of pro-criminal and pro-violence attitudes e.g., [32]. These cognitions are labeled “neutralizations”, which comprise justifications of, excuses for, and denials of wrongdoing. Law violators can believe in law-abiding norms while still violating them, preserving their self-image by generating reasons for their actions. These cognitions, also commonly regarded as cognitive distortions, are associated with various forms of deviant and criminal behavior [33]. According to the general aggression model [34], a recent and prominent theoretical model of violent behavior, cognitions are “inputs” that increase an individual’s propensity to behave violently; these cognitions include factors such as attitudes and beliefs. Thus, pro-violence attitudes are considered a key contributor to violent responses. These cognitive inputs are likely to influence an individual’s internal state (i.e., thoughts, feelings, and arousal) and the automatic inferences they make about social encounters. Studies have generally found an inverse relationship between perceptions of overall police legitimacy and public attitudes toward the acceptability of violence, wherein positive judgments about police legitimacy are associated with relatively negative views of the use of violence, particularly among younger generations [35,36,37].

Over the last few decades, various theoretical approaches have been proposed to explain an individual’s mental well-being. The relationship between an individual’s temperament and pro-criminal attitudes and behaviors is well supported by the literature. In the criminological context, strain theories have been widely used to elucidate the influence of negative emotions or poorer mental health on the tendency to adopt deviant attitudes and/or behavior. General strain theory [38] hypothesizes that experiencing strains or stressors may interact with individual characteristics to amplify the risk of adopting maladaptive attitudes such as pro-criminal and pro-violence attitudes and behaviors, including criminal activity, aggression, and violence. These manifestations of negative coping are thus in response to antagonistic incidents, conditions, or treatment. Negative emotions such as depression, anger, or frustration act as stimuli for action that triggers a progression from the experience of strain to deviant attitudes and offending behavior [39].

According to this theoretical perspective, deviant attitudes and behaviors are corrective actions taken to condemn, injure, or retaliate against the presumed sources of strain. Felson’s [40] social interactionist approach postulates that an individual’s negative affects, such as resentment, aggression, or desire for revenge may lead to pro-violence attitudes, eventually creating a cycle of violence and retaliation. Retaliation is likely to cause an escalation in violence [41]. Few studies have examined the relationship between negative affects and perceived police procedural and distributive justice. Nonetheless, the general findings of these studies indicate a positive link between perceived procedural injustice exhibited by police (police-related strain; e.g., police officers’ dishonesty, discourtesy, disrespectful behavior, and discrimination against minorities) and negative emotions or poor mental well-being (e.g., anger, frustration, resentment), which subsequently increase the likelihood of criminal offending [42,43,44,45,46].

Self-control theory, also known as the general theory of crime, is one of the most tested and well-supported explanations of deviant attitudes and offending behaviors [47]. According to Gottfredson and Hirschi [48], individuals with less self-control are more likely to adopt deviant attitudes and to become involved in delinquent or criminal activities in pursuit of immediate gratification without considering the potential consequences. Individuals with low self-control tend to be self-centered, risk-seeking, impulsive, and short-tempered, and prefer physical over mental activities and simple over complex tasks [49]. Forming between the ages of 6 and 10 [50], these six key personality traits are likely to remain extremely stable over the life course, regardless of demographic characteristics such as age, sex, culture, and social class [51]. The developmental phase of individuals who lack self-control is often characterized by early exposure to criminogenic environments that preclude the development of sufficient control over one’s behavior. Consistent with theoretical assertions [48], Wolfe’s [52] study of college students found that low levels of self-control were significantly associated with negative judgments of police procedural justice. The effect of perceived procedural justice on perceived police legitimacy is weakened when levels of self-control are lower (see also [53]).

### The Present Study

Focusing on a large sample of Hong Kongers and drawing on important mainstream theories, this study explored several psychosocial factors (i.e., attitudes toward violence, general mental health, and self-control) that were potentially correlated with general perceptions of police procedural and distributive justice, specifically during and immediately after the 2019–2020 Anti-ELAB Movement. This study may be the first empirical work of its kind. Most importantly, the findings of this study offer insights for practitioners (e.g., public educators and policy developers) by identifying significant psychosocial factors associated with a person’s likelihood of holding negative perceptions of general, procedural, and distributive fairness of the police. Timely and effective interventions such as pragmatic precautionary measures and public education are essential to enhance individuals’ perceptions of police procedural and distributive justice, as well as their sense of obligation to follow legal directives and rules. This is particularly important during the current period of social instability in Hong Kong. Such positive perceptions help to reduce public engagement in anti-government activities, shape social norms, and foster compliance with the law, thus promoting public safety, socio-economic development, and social stability in Hong Kong. Drawing on the literature, the following research hypotheses are put forward.

**Hypothesis** **1** **(H1).** *There are sex and age-group differences in the mean levels of police general, procedural, and distributive justice and psychosocial factors (i.e., general mental health, attitudes toward violence, and self-control).*

**Hypothesis** **2** **(H2).** *Psychosocial factors are associated with Hong Kongers’ perceptions of police general, procedural, and distributive justice, even when controlling for their age group (i.e., young adults vs. middle-aged and older adults) and demographic characteristics (i.e., age, sex, religiosity, marital status, political inclination, satisfaction with experiences with the police, and trust in the government), in that high levels of general mental health and self-control and low tolerance of violence are associated with positive perceptions of police general, procedural, and distributive justice.*

## 3. Methods

### 3.1. Participants and Procedure

Hong Kong is a modern semi-autonomous city (as a Special Administrative Region of the PRC). Approximately 95% of its residents are of Chinese descent. Since 1 July 1997, Hong Kong has been a major financial hub in the Asia-Pacific region. The influence of over 150 years of British colonization (1842–1997) means that Hong Kongers generally balance a modern Western lifestyle with traditional Chinese cultural values and practices. Similar to other former British colonies, Hong Kong has a criminal justice system based on the U.K.’s common law system, which largely emphasizes the rule of law and due process [54].

This study recruited 1024 Hong Kong residents who were aged at least 18 and had remained in Hong Kong for most of the social unrest in the second half of 2019 to participate in a questionnaire. The data collection was conducted between April and September 2020. To ensure that the sample was as representative as possible, these participants were recruited from different populations. The first pool of participants was randomly recruited from various secondary and post-secondary education institutions (e.g., vocational education institutes and universities) in Hong Kong. The second group comprised Hong Kongers who were not pursuing education at the time of the study (e.g., working adults, housewives, retirees, and the unemployed) but who may have previously received secondary and/or post-secondary education. A randomly convenience sampling approach was used to recruit the second group of participants, targeting an equal number of middle-to-high- and low-income communities in various districts. The participants were recruited from the entrance surroundings of public and private housing, as well as community service centers. Upon giving their informed consent, the participants filled out the questionnaire either on paper or online (i.e., a Qualtrics Survey). They were assured that their participation in the study was completely voluntary, and that their responses would be kept confidential and used only for research purposes. An average of 25 min was required to complete the questionnaire. The response rate and cooperation rate for the survey were each approximately 90%.

Of the 1024 participants, 66.4% were females (n = 680) and the remaining 33.6% were males (n = 344; see Table 1). The average age was 29.15 (*SD* = 9.28, range = 18–69), with no significant differences in age between the sexes (males: *M* = 29.66, *SD* = 9.62; females: *M* = 28.89, *SD* = 9.10). More than two-thirds of the participants (68.3%) were young adults (18 to 30 years old) and the rest (31.7%) were middle-aged and older adults (31 years and above). The Hong Kong population estimated by the Census and Statistics Department at the end of 2020 was as follows: 15–19 years old (3.6%), 20–29 years old (11.2%), 30–39 years old (15.3%), 40–49 years old (15.4%), 50–59 years old (16.0%), and 60–69 years old (14.3%) [55]. A large majority of the participants were Hong Kongers (87.7%), and just over half (59.7%) reported as non-single. Nearly two-thirds of the participants (63.6%) reported no religious affiliation, and approximately three-quarters (74.6%) had received post-secondary education. Just over three-quarters of the participants (76.2%) claimed that they supported either the pro-democracy camp or the pro-independence/radical democracy political camp, and the majority declared themselves to be Hong Kong citizens (89.5%). A self-reported record of police arrest was uncommon (3.8%) among the participants in this sample.

### 3.2. Measures

Self-report measures were adopted to examine the following: (1) the overall means, along with the sex and age differences in these means, for the participants’ perceptions of police general, procedural, and distributive justice; and psychosocial factors (i.e., general mental health, attitudes toward violence, and self-control); and (2) the age effects of psychosocial factors in association with the participants’ perceptions of police general, procedural, and distributive justice, while controlling for their demographic characteristics. The questionnaire was printed in both English and Chinese versions for participants with different language needs. The scales were written in English and translated into Chinese by an experienced translator with appropriate academic qualifications. The Chinese version was then back-translated into English to ensure face validity and for comparison with the original English written scales to ensure content similarity.

#### 3.2.1. Perceptions of Police Procedural and Distributive Justice Scale

A five-item measure was used to explore the participants’ perceptions of police procedural and distributive justice, consisting of two sub-scales measuring procedural and distributive justice. The items were measured on a 5-point Likert scale (0 = *strongly disagree*, 4 = *strongly agree*), with the total score ranging from 0 to 20.

Perceived police procedural justice is broadly defined as the perceived fairness of the procedures followed by the police. It is a key determinant of citizens’ trust and confidence in the police and police performance [56]. The procedural justice sub-scale, with three items, was used to assess the participants’ general views of how Hong Kong’s police force treats citizens [5]. The total score for this sub-scale ranged from 0 to 12 points, with higher scores indicating more positive perceptions of procedural fairness. Sample items included “Police are concerned about respecting a citizen’s individual rights” and “Police treat people as if they can be trusted to do the right thing”.

Based on the assumption that authorities such as the police are expected to fairly distribute services across people and communities, a two-item distributive justice sub-scale was used to measure the participants’ opinions on whether police officers offer preferential treatment to certain groups of people [5]. The total score ranged from 0 to 8 points, with higher scores denoting more positive perceptions of distributive fairness. A sample item was “It’s about not what you’ve done but who you are and who you know when it comes to the police”. Cronbach’s α for this measure was 0.70 (males = 0.72, females = 0.69; young adults = 0.70, middle-aged and older adults = 0.70), with the alpha coefficient values of the procedural and distributive justice sub-scales being 0.78 (males = 0.75, females = 0.79) and 0.63 (males = 0.66, females = 0.61), respectively. Although the inter-item reliability of the distributive justice subscale was lower than the preferred Cronbach’s α level of 0.70 [57], Streiner and Norman [58] (p. 64) argued that Cronbach’s α is “dependent not only on the magnitude of the correlations among items, but also on the number of items”.

#### 3.2.2. General Mental Questionnaire

A 12-item general health questionnaire (GHQ-12) was used to measure the participants’ general mental health in the previous 12 months [59]. Responses to each item were given in a dichotomous format (0 = *no*, 1 = *yes*), with a higher score indicating greater positive general mental health. The total score ranged from 0 to 12 points. Sample items included “Felt capable of making decisions about things” and “Was able to enjoy normal day-to-day activities”. The internal consistency of this measure was 0.70 (males = 0.69, females = 0.71; young adults = 0.68, middle-aged and older adults = 0.73).

#### 3.2.3. Attitudes toward Violence Scale

The 15-item Attitudes Toward Violence Scale (ATVS) proposed by Funk et al. [30] was adopted to assess the participants’ attitudes toward violent behavior. The items were assessed on a 5-point Likert scale (1 = *strongly disagree*, 5 = *strongly agree*). The total score ranged from 15 to 75 points, with higher scores reflecting greater acceptance of violence. Sample items included “If a person hits you, you should hit them back” and “It’s okay to use violence to get what you want”. In this study, Cronbach’s α for the ATVS was 0.87 (males = 0.87, females = 0.87; young adults = 0.86, middle-aged and older adults = 0.89).

#### 3.2.4. Low Self-Control Scale

Based on Gottfredson and Hirschi’s [48] self-control theory, a 23-item Low Self-Control Scale (LSCS) [60] was adopted to measure the participants’ levels of self-control. This scale measures the six self-control elements (impulsivity, volatile temper, self-centeredness, risk-seeking, preference for simple (vs. complex) tasks, and preference for physical (vs. mental) activities) that are widely regarded as indicators of low self-control. Items on this scale were assessed on a 4-point Likert scale (1 = *strongly agree*, 4 = *strongly disagree*), with the total score ranging from 23 to 92 points. Higher scores indicated greater self-control. Sample items included “Sometimes I take risks just for the fun of it” and “I always do whatever brings me pleasure here and now, even at the cost of some distant goal”. The internal consistency of the LSCS in this study was 0.89 (males = 0.91, females = 0.89; young adults = 0.88, middle-aged and older adults = 0.91).

### 3.3. Analytic Strategy

Independent-samples *t*-tests were first computed to assess the sex and age-group differences in the means of the participants’ general perceptions of police procedural and distributive justice, as well as psychosocial factors. Next, a series of ordinary least squares (OLS) regressions were performed to explore the age-group effects of various psychosocial factors on general perceptions of police procedural and distributive justice, while controlling for the participants’ demographic characteristics (i.e., age, sex, religiosity, marital status, police arrest record, political affiliation, satisfaction with experiences with the police, and trust in the government). The OLS regression model was adopted because it permits both estimation of the values of a continuous response variable using more than one explanatory variable and identification of the strength of the relationships between these variables. The participants’ religiosity was measured using a 6-point Likert scale asking how religious they perceived themselves to be (1 = *not at all*, 6 = *very*). One item each was used to assess the participants’ satisfaction with police services and their trust in the government. Responses were given on a 5-point Likert scale (0 = *strongly disagree*, 4 = *strongly agree*), with higher scores reflecting greater satisfaction with the police and trust in the government. Pearson correlations of the tested variables were computed. No correlation at or above 0.70 was found, indicating no collinearity. The significance level was set at 0.05. Other OLS assumptions (i.e., normality of independent and dependent variables, absence of outliers, homoscedasticity, and residuals’ independence) were also tested, and no violations were found. No data imputation was performed on the missing data; as the proportion of missing data was small (smaller than 10%), they were regarded as missing at random.

### 3.4. Ethical Considerations

This study was approved by the ethical committee of the author’s university. The participants were allowed to leave the study, contact the primary investigator, and/or access professional counseling at any time. The data were collected anonymously, with no personally identifying details recorded.

## 4. Results

### 4.1. Sex and Age-Group Differences in Perceived Police Procedural and Distributive Justice and Psychosocial Factors

The mean differences in perceived police procedural and distributive justice and psychosocial factors between the male and female participants and between the young adult and the middle-aged and older adult participants are presented in Table 2 (Hypothesis 1). Relative to the female participants, the male participants reported significantly more positive general perceptions of police procedural and distributive justice (*t* = −1.87, *p* = 0.049). In terms of psychosocial factors, the male participants similarly scored significantly higher for general mental health (*t* = −3.04, *p* = 0.002) and reported significantly more negative attitudes toward violence (*t* = −3.40, *p* = 0.001) than their female counterparts did. In terms of age-group differences, the young adults reported significantly higher scores than the middle-aged and older adults regarding general perceptions of police procedural and distributive justice (*t* = 3.93, *p* < 0.001), procedural fairness (*t* = 2.19, *p* = 0.029), and distributive fairness (*t* = 3.31, *p* = 0.001). Relative to the young adults, the middle-aged and older adults reported significantly better general mental health (*t* = −6.57, *p* < 0.001) and self-control (*t* = −4.19, *p* < 0.001).

### 4.2. Effects of Psychosocial Factors on Perceptions of Police General, Procedural, and Distributive Justice by Age Group

OLS regressions were performed to explore the effects of the psychosocial factors on the participants’ perceptions of police general, procedural, and distributive justice, while controlling for their demographic characteristics (Hypothesis 2). Table 3 shows that all of the regression models were significant. In general, the participants’ self-control (overall sample: *B* = 0.04, *SE* = 0.01, *p* < 0.001; young adults: *B* = 0.02, *SE* = 0.01, *p* = 0.034; middle-aged and older adults: *B* = 0.07, *SE* = 0.02, *p* < 0.001) was significantly associated with their general perceptions of police procedural and distributive justice, regardless of their age. In addition to the significant findings noted in the overall sample, general mental health (young adults: *B* = 0.10, *SE* = 0.04, *p* = 0.006; overall sample: *B* = 0.07, *SE* = 0.03, *p* = 0.015) and attitudes toward violence (young adults: *B* = −0.03, *SE* = 0.01, *p* = 0.003; overall sample: *B* = −0.03, *SE* = 0.01, *p* = 0.004) were only significantly correlated with positive general perceptions of police procedural and distributive justice among young adults. Several characteristics were significant predictors for all of the participants: affiliation with the pro-establishment political camp (overall sample: *B* = −0.94, *SE* = 0.19, *p* < 0.001; young adults: *B* = −0.84, *SE* = 0.24, *p* < 0.001; middle-aged and older adults: *B* = −1.19, *SE* = 0.32, *p* < 0.001); a high level of satisfaction with the police (overall sample: *B* = 1.38, *SE* = 0.13, *p* < 0.001; young adults: *B* = 1.30, *SE* = 0.17, *p* < 0.001; middle-aged and older adults: *B* = 1.54, *SE* = 0.23, *p* < 0.001); and a high level of trust in the government (overall sample: *B* = 0.86, *SE* = 0.14, *p* < 0.001; young adults: *B* = 0.99, *SE* = 0.17, *p* < 0.001; middle-aged and older adults: *B* = 0.53, *SE* = 0.23, *p* = 0.021). In contrast, being more religious was significantly associated with general positive perceptions of police procedural and distributive in the overall sample (*B* = 0.4, *SE* = 0.05, *p* = 0.010) and among middle-aged and older adults (*B* = 0.33, *SE* = 0.10, *p* = 0.001), while being non-single was only found to be significant among middle-aged and older adults (*B* = 0.72, *SE* = 0.32, *p* = 0.025). Nevertheless, being male (*B* = 0.16, *SE* = 0.34, *p* = 0.033) was only found to be a significant predictor in the overall sample.

Specifically, tolerance of violence (overall sample: *B* = −0.03, *SE* = 0.01, *p* < 0.001; young adults: *B* = −0.02, *SE* = 0.01, *p* = 0.002; middle-aged and older adults: *B* = −0.05, *SE* = 0.01, *p* < 0.001) was significantly correlated with perceived procedural fairness regardless of the participants’ age, while self-control was a significant predictor in the overall sample (*B* = 0.02, *SE* = 0.01, *p* = 0.005) and among young adults (*B* = 0.02, *SE* = 0.01, *p* = 0.030). Demographically, having high levels of satisfaction with the police (overall sample: *B* = 1.17, *SE* = 0.09, *p* < 0.001; young adults: *B* = 1.11, *SE* = 0.11, *p* < 0.001; middle-aged and older adults: *B* = 1.28, *SE* = 0.15, *p* < 0.001) and trust in the government (overall sample: *B* = 0.87, *SE* = 0.09, *p* < 0.001; young adults: *B* = 1.01, *SE* = 0.12, *p* < 0.001; middle-aged and older adults: *B* = 0.59, *SE* = 0.16, *p* < 0.001) were significant predictors across all of the participants. However, being non-single was only significantly associated with positive perceptions of police procedural fairness in the overall sample (*B* = −0.24, *SE* = 0.11, *p* = 0.031) and among young adults (*B* = −0.29, *SE* = 0.13, *p* = 0.025). Similarly, being affiliated with the pro-establishment political camp was only found to be a significant predictor in the overall sample (*B* = −0.33, *SE* = 0.13, *p* = 0.010) and among young adults (*B* = −0.40, *SE* = 0.16, *p* = 0.013).

Regardless of the participants’ age, perceptions of police distributive fairness were associated with their attitudes toward violence (overall sample: *B* = −0.06, *SE* = 0.01, *p* < 0.001; young adults: *B* = −0.06, *SE* = 0.01, *p* < 0.001; middle-aged and older adults: *B* = −0.06, *SE* = 0.01, *p* < 0.001) and self-control (overall sample: *B* = 0.06, *SE* = 0.01, *p* < 0.001; young adults: *B* = 0.04, *SE* = 0.01, *p* < 0.001; middle-aged and older adults: *B* = 0.09, *SE* = 0.01, *p* < 0.001). General mental health was significantly correlated with positive perceptions of police distributive fairness in the overall sample (*B* = 0.07, *SE* = 0.02, *p* = 0.003) and among young adults (*B* = 0.12, *SE* = 0.03, *p* < 0.001). An affiliation with the pro-establishment political camp (overall sample: *B* = 0.61, *SE* = 0.16, *p* < 0.001; young adults: *B* = −0.43, *SE* = 0.21, *p* = 0.036; middle-aged and older adults: *B* = −0.94, *SE* = 0.28, *p* = 0.001) was the only significant predictor of perceived police distributive fairness among all of the participants. Being more religious and being single were significant predictors of positive perceptions of police distributive fairness in the overall sample (religiosity: *B* = 0.12, *SE* = 0.05, *p* = 0.016; marital status: *B* = 0.28, *SE* = 0.14, *p* = 0.043) and among middle-aged and older adults (religiosity: *B* = 0.30, *SE* = 0.08, *p* < 0.001; marital status: *B* = 0.67, *SE* = 0.27, *p* = 0.013). Being male (*B* = 0.36, *SE* = 0.17, *p* = 0.036) was only found to be significantly associated with positive perceptions of police distributive fairness among young adults, while having a police arrest record (*B* = 0.71, *SE* = 0.34, *p* = 0.040) was only found to be significant in the overall sample.

## 5. Discussion

Empirical research has consistently demonstrated that perceptions of police procedural and distributive justice can promote citizens’ cooperation and compliance with the police, as well as their general willingness to obey the law. A systematic review by Mazerolle and colleagues [61] found that when police use fair procedures, treat citizens with respect and dignity, and give citizens a voice during police–citizen interactions, citizen compliance increases. A trusting relationship between police and citizens is likely to improve the crime control function of the police through citizens’ perceived obligation to obey the law and consequent law-abiding behaviors [62]. In contrary, the perceived police illegitimacy can further radicalize protestors as a result of both the structural context and the intergroup dynamics partly formed by coercive policing practices [20]. This study not only advanced knowledge of this topic with a rarely researched sample drawn from a large pool of Hong Kongers, but also indicated practical ways to improve public perceptions of police procedural and distributive justice. The primary objectives of this study were: (1) to explore the relationships between psychosocial factors and perceptions of police procedural and distributive justice; and (2) to investigate whether these relationships held when controlling for the participants’ age group and demographic characteristics. In general, the male participants reported significantly more positive general perceptions of police procedural and distributive justice, better general mental health, and more negative attitudes toward violence than the female participants did. Consistent with the findings in [17], young participants reported significantly more positive general perceptions of police procedural and distributive justice than their middle-aged and older counterparts did. However, relative to young adults, middle-aged and older adults scored significantly higher for general mental health and self-control. Hypothesis 1 was largely supported.

A number of findings regarding the effects of psychosocial factors on the participants’ willingness to cooperate with the police warrant further discussion. These findings supported some of the major criminological theories explaining public perceptions of police procedural and distributive justice. Of note, Hypothesis 2 was only partially supported. It is interesting to note the significant influence of attitudes toward violence on general perceptions of police procedural and distributive justice across ages, with the sole exception of middle-aged and older adults’ general perceptions of police procedural and distributive justice. Put simply, those who adopted pro-violence attitudes were less likely to perceive the police as operating under fair and equitable procedures and treating everyone fairly. This was consistent with the previous finding that a pro-violence attitude was associated with a tendency to avoid cooperating with the police [2,35,36,37].

Another significant finding was the effect of self-control on the participants’ perceptions of police legitimacy. Across ages, the participants’ levels of self-control were significantly correlated with their perceptions of general, procedural, and distributive fairness (with the exception of the relationship between self-control and perceived police procedural fairness for middle-aged and older adults). The low self-control theoretical proposition was supported in this study, in that higher levels of self-control were found to be significantly associated with positive perceptions of police general procedural and distributive justice. Conversely, a lack of self-control has long been found by cross-cultural studies to predict deviant attitudes and behavior (e.g., [47,63]). These studies indicated that individuals with low self-control tend to be impulsive and self-centered, engaging in risky behavior and seeking immediate benefits without considering potential long-term adverse consequences. In terms of the present study’s focus, negative perceptions of police procedural and distributive fairness were likely to encourage an individual to be uncooperative and noncompliant with the police, leading to a tendency to engage in illegal activities.

Consistent with the limited literature in the area (e.g., [42,43,45,46]), this study found a significant positive relationship between mental health (e.g., positive emotions) and general perceptions of police procedural and distributive justice. Conversely, perceived violations of procedural fairness led to feelings of strain. The violation of public expectations of how police should behave may erode people’s commitment to norms in terms of respect for the police and compliance with the law [44]. However, this significant relationship was only noted among young adults. Communication between police and young (vs. middle-aged and older) people, particularly at-risk or marginal young people, is more likely to contain elements of interactional injustice, which can subsequently lead to perceptions of strain [43]. This form of perceived injustice mainly reflects the quality of the treatment that an individual receives when interacting with the police; i.e., perceived distributive fairness (or interactional injustice). Negative emotions such as anger, resentment, bitterness, and depression are likely to be generated by perceived interactional injustice, which in turn can have negative outcomes such as deviant attitudes and criminal behavior [64].

There were several interesting findings concerning the participants’ demographic characteristics. First, a reported affiliation with the pro-establishment political camp was associated with positive perceptions of police general, procedural, and distributive justice. Put differently, participants with this affiliation (vs. a pro-democracy/pro-independence affiliation) were more likely to be supportive of the police and less inclined to be involved in anti-government activities, including demonstrations. These findings were supported by the fact that the Anti-ELAB Movement was mainly initiated by the pro-democracy and pro-independence camp [65]. Furthermore, participants of all ages who were satisfied with their experiences of dealing with the police and reported more trust in the government were significantly more likely to report positive perceptions of police general and procedural justice, but not police distributive fairness. They were more inclined to regard the police as procedurally legitimate and fair. According to de Lange and colleagues’ [66] cognitive science study, prior knowledge, including experience, has a profound impact on the ways in which people perceive the world. In the context of the present study, positive or satisfactory of past experiences with the police and general trust in authority can foster positive perceptions of and support for the police.

Several methodological limitations in this study require attention. One limitation of this study was its correlational nature; at most, the findings can only be explained in correlational terms. Future research could consider adopting a longitudinal design to better comprehend the causal relationships between the participants’ psychosocial factors and their perceptions of police general, procedural, and distributive justice. Furthermore, other factors may confound the associations between significant psychosocial factors and general perceptions of police procedural and distributive justice. Future research could test the underlying mechanisms of the target associations, such as the potentially mediating roles of the variables included in this study’s regression models or other theoretically relevant variables. Second, this study was limited to the use of self-reported data. Cognitive biases, such as retrospective recall bias and social desirability bias, may have influenced the participants’ truthfulness in reporting their perceptions, attitudes, and experiences. A measure of response bias should be considered in future studies to address the limitations of using self-reported data. Third, the measure of general perceptions of police procedural and distributive in this study included only a five-item scale assessing perceived procedural and distributive justice. Scales that explore general perceptions of police legitimacy in terms of legality and effectiveness, as suggested by Tankebe [67], were not used in this study. In addition, adopting a scale that only consisted of five items may not have been sufficient enough to capture and a more comprehensive conceptualization of perceived police procedural and distributive justice. Hence, future studies that examine the perceptions of police legitimacy should include measures of police legality and effectiveness in addition to procedural and distributive justice. In terms of the sampling population, two-thirds of the participants recruited in this study were females, and nearly 70% were young adults aged 18 to 30. Thus, the findings are not necessarily generalizable to the wider Hong Kong population, particularly older Hong Kongers. Future studies should recruit participants from all walks of life. Nevertheless, this sample was representative of Hong Kong’s younger population.

### Implications of the Findings

Consistent with previous research on public perceptions of police procedural and distributive justice, this study’s findings have important implications in terms of public education and policy development/refinement, especially with regard to strategically and effectively engaging Hong Kongers in initiatives that will enhance their overall perceptions of police procedural and distributive justice, and encourage them to support the police. Such interventions are particularly essential for young people. School education programs focused on promoting prosocial functioning (e.g., avoiding attitudes supportive of violence, improving mental well-being, reducing negative emotions, and enhancing self-control) should be developed and should involve parents, school administrators, teachers, and social-service providers. Studies have consistently found that parental involvement has a significant effect on adolescents and young adults’ future involvement in deviant and criminal activities, both as offenders and as victims (e.g., [29]). When necessary, social-service providers (e.g., social workers) can act as an effective bridging agent between parents and their children when tension arises between the two parties. Furthermore, community-based resources and counseling services to promote healthy and prosocial self-development should be strengthened to support individuals in need of professional help to cope with their problems (e.g., negative emotionality). Such resources may include public mental health seminars (with topics such as anger management and self-assertiveness) and social norm interventions (e.g., to correct the misperception that violence is an effective problem-solving method).

In terms of policy implications, police procedural and distributive justice relies heavily on whether the police effectively accomplish their traditional functions (e.g., rapid response, crime prevention and solving, and victim assistance) and whether members of the public are treated fairly in police encounters. Unsatisfying encounters with the police are likely to have both normative and instrumental costs for individuals, including weakening their perceptions of police procedural and distributive justice. Stott and colleagues [20] found that police inaction during critical moments and the use of coercive policing practices were likely to anger the protestors, who subsequently became “radicalized”. This can reduce police officers’ effectiveness in performing their crime-control duties. The success of the police depends heavily on citizens’ willingness to cooperate. Thus, building positive police–citizen relationships is essential, particularly among young people. Research has found that young people’s negative perceptions of and attitudes toward the police, which are often formed during adolescence, endure into adulthood [68]. As such, negative attitudes toward the police are likely to negatively influence their compliance with police requests and with the law in general [4]. This observation was particularly relevant to this study, as the majority of the participants recruited were young people.

Given that low levels of self-control are associated with negative perceptions of police procedural and distributive fairness, it is important for the police to be aware that compliance obtained from individuals with low self-control may not be genuine; they may comply with police requests simply to serve their own interests (e.g., to avoid being arrested at that moment) [52]. These individuals may be concerned primarily about their immediate well-being and gratification; they are unlikely to intend to cooperate with the police in the long term. Hence, compliance is unlikely to reflect lasting impressions or perceptions of police procedural and distributive justice among individuals who lack self-control. This may also be true for those with poorer mental health or more negative emotions (e.g., anger, resentment, or frustration) and those whose who tend to adopt pro-violence attitudes (e.g., aggression). Genuine support for and cooperation with the police may be difficult to obtain from these individuals. Carefully explaining to these individuals how to avoid arrest by appealing to self-centered and anti-social aspects of their personalities may be a practical approach. To promote lasting positive perceptions of police procedural and distributive fairness, the police should be aware of situations in which their patience may be tested and they may be provoked to act in procedurally unjust ways. Therefore, officers may benefit from emotional management and other prosocial training, such as anger management and victim empathy workshops, to enhance their psychosocial functioning. Basic de-escalation training that emphasizes effective communication and active listening skills helps police officers handle crisis situations [69]. To enhance public perceptions of procedural and distributive fairness, Hong Kong’s police officers should stay neutral and transparent in their decision making, show genuine concern for the interests of all parties, treat citizens from different backgrounds fairly and with respect, and where appropriate, involve citizens in the decision-making process.

## 6. Conclusions

Despite the noted shortcomings, this study has provided an important step forward in better understanding public perceptions of police procedural and distributive justice, particularly during and immediately after the 2019–2020 Anti-ELAB Movement. Given the current volatile political situations in Hong Kong, this study is pertinent and timely to empirically demonstrating the importance of psychosocial factors (i.e., general mental health, attitudes toward violence, and self-control) in an individual’s likelihood of holding negative perceptions of general, procedural, and distributive fairness of the police. Understanding the significant psychosocial factors that may play a role in their perceived police procedural and distributive justice may help to identify risk factors for purposes of pragmatic precautionary measures and public education. The findings of this study further support the applicability of major criminological theories in explaining public perceptions of police procedural and distributive fairness in the Hong Kong Chinese context. Future studies that sample non-Western populations should be encouraged in order to identify the role of cultural and political factors in influencing an individual’s perceptions of general, procedural, and distributive fairness of the police, and to advance our theoretical knowledge through cross-cultural research.

## Figures and Tables

**Table 1:** Sample demographic characteristics (*N* = 1024).

Variable	*N*	Percentage
Sex		
Male	344	33.6%
Female	680	66.4%
Age group		
Young adult (18–30 years old)	699	68.3%
Middle-aged and older adult (31 years and above)	325	31.7%
Place of origin		
Hong Kong	898	87.7%
Mainland China	114	11.1%
Others (e.g., Macau, Taiwan, India, Canada, and USA)	12	1.2%
Marital status		
Non-single	612	59.7%
Single	412	40.3%
Religious belief		
Without a religious belief	651	63.6%
With a religious belief	373	36.4%
(e.g., Christianity, Catholic, Buddhism, Muslim, Sikhism)		
Highest education attainment		
Primary school education	7	0.7%
Secondary school education	253	24.7%
Post-secondary school education	764	74.6%
(e.g., associate degree/high diploma; and undergraduate and post-graduate degrees)		
Political inclination		
Pro-democracy and pro-independence camp	780	76.2%
Non-pro-democracy and non-pro-independence camp	244	23.8%
(e.g., pro-establishment, centralist, and politically neutral)		
Self-perceived identity		
Hong Kong citizen	916	89.5%
Chinese citizen	22	2.1%
Hong Kong Chinese citizen	23	2.2%
Chinese Hong Kong citizen	44	4.3%
Others (e.g., HK permanent resident with other citizenship)	19	1.9%
Self-reported police arrest		
Yes	39	3.8%
No	984	96.2%

**Table 2:** Sex and age-group differences of the prevalence of perceptions of police procedural and distributive justice, as well as psychosocial characteristics.

										Middle-Aged and		
	Overall Sample		Female		Male			Young Adults		Older Adults		
	(*N* = 1024)		(n = 680)		(n = 344)			(n = 699)		(n = 325)		
Variable	*M*	*SD*	*M*	*SD*	*M*	*SD*	*t* Value	*M*	*SD*	*M*	*SD*	*t* Value
General perceptions	18.11	3.64	17.96	3.69	18.41	3.54	−1.87 *	18.41	3.61	17.45	3.63	3.93 ***
Procedural justice	12.01	3.02	11.93	3.04	12.17	2.96	−1.19	12.15	3.03	11.71	2.96	2.19 *
Distributive justice	6.10	2.25	6.03	2.20	6.24	2.34	−1.44	6.26	2.18	5.75	2.37	3.31 **
Psychosocial characteristics												
General mental health	5.89	2.78	5.70	2.78	6.26	2.74	−3.04 **	5.51	2.66	6.71	2.86	−6.57 ***
Attitudes toward violence	42.70	9.39	42.00	9.25	44.10	9.53	−3.40 **	42.72	8.91	42.68	10.38	0.06
Self-control	65.50	9.11	65.73	8.80	65.04	9.71	1.14	64.69	8.86	67.25	9.43	−4.19 ***

* *p* < 0.05, ** *p* < 0.01, *** *p* < 0.001.

**Table 3:** OLS regression models of the participants’ perceptions of police procedural and distributive justice.

	General Perceptions of Police Procedural and Distributive Justice						Perceived Police Procedural Justice					
	Overall Sample		Young Adults		Middle-Aged and Older		Overall Sample		Young Adults		Middle-Aged and Older	
	(*N* = 1024)		(n = 699)		Adults (n = 325)		(*N* = 1024)		(n = 699)		Adults (n = 325)	
Predictors	*B*	(*SE*)	*B*	(*SE*)	*B*	(*SE*)	*B*	(*SE*)	*B*	(*SE*)	*B*	(*SE*)
Demographic characteristics												
Age	−0.01	0.01					−0.01	0.01				
Sex (1 = male)	0.34	0.16 *	0.35	0.20	0.43	0.28	0.08	0.11	−0.01	0.14	0.24	0.19
Religiosity	0.14	0.05 *	0.04	0.07	0.33	0.10 **	0.03	0.04	0.01	0.05	0.02	0.07
Marital status (1 = single)	0.05	0.16	−0.17	0.19	0.72	0.32 *	−0.24	0.11 *	−0.29	0.13 *	0.04	0.21
Police arrest record	0.49	0.40	0.44	0.58	0.32	0.54	−0.22	0.27	−0.21	0.40	−0.33	0.36
Political inclination (1 = pro-democracy and pro-independence)	−0.94	0.19 ***	−0.84	0.24 ***	−1.19	0.32 ***	−0.33	0.13 *	−0.40	0.16 *	−0.25	0.22
Satisfied experience with the police	1.38	0.13 ***	1.30	0.17 ***	1.54	0.23 ***	1.17	0.09 ***	1.11	0.11 ***	1.28	0.15 ***
Public trust in the government	0.86	0.14 ***	0.99	0.17 ***	0.53	0.23 *	0.87	0.09 ***	1.01	0.12 ***	0.59	0.16 ***
Psychosocial factors												
General mental health	0.07	0.03 *	0.10	0.04 **	0.02	0.05	0.01	0.02	0.03	0.02	0.04	0.03
Attitudes toward violence	−0.03	0.01 **	−0.03	0.01 **	−0.01	0.02	−0.03	0.01 ***	−0.02	0.01 **	−0.05	0.01 ***
Self-control	0.04	0.01 ***	0.02	0.01 *	0.07	0.02 ***	0.02	0.01 **	0.02	0.01 *	0.02	0.01
Constant	11.80	0.70 ***	11.08	0.82 ***	12.18	1.19 ***	0.67	0.48 *	0.58	0.56 *	0.77	0.80 *
Adjusted R^2^	0.58		0.57		0.60		0.72		0.72		0.72	
*F*	128.75 ***		94.21 ***		48.01 ***		237.01 ***		178.18 ***		84.54 ***	
	**Perceived Police Distributive Justice**											
	**Overall Sample**			**Young Adults**				**Middle-Aged and Older**				
	**(*N* = 1024)**			**(n = 699)**				**Adults (n = 325)**				
**Predictors**	***B***	**(*SE*)**		***B***		**(*SE*)**		***B***		**(*SE*)**		
Demographic characteristics												
Age	0.01	0.01										
Sex (1 = male)	0.27	0.14		0.36		0.17 *		0.20		0.24		
Religiosity	0.12	0.05 *		0.03		0.06		0.30		0.08 ***		
Marital status (1 = single)	0.28	0.14 *		0.12		0.16		0.67		0.27 *		
Police arrest record	0.71	0.34 *		0.65		0.51		0.64		0.46		
Political inclination (1 = pro-democracy and pro-independence)	0.61	0.16 ***		−0.43		0.21 *		0.94		0.28 **		
Satisfied experience with the police	0.21	0.12		0.20		0.14		0.26		0.19		
Public trust in the government	−0.02	0.12		−0.02		0.15		−0.05		0.20		
Psychosocial factors												
General mental health	0.07	0.02 **		0.12		0.03 ***		0.02		0.04		
Attitudes toward violence	−0.06	0.01 ***		−0.06		0.01 ***		−0.06		0.01 ***		
Self-control	0.06	0.01 ***		0.04		0.01 ***		0.09		0.01 ***		
Constant	11.14	0.61 ***		10.50		0.71 ***		11.41		1.02 ***		
Adjusted R^2^	0.18			0.12				0.30				
*F*	20.81 ***			10.43 ***				14.81 ***				

Note: unstandardized beta (B) and standard error (SE). * *p* < 0.05, ** *p* < 0.01, *** *p* < 0.001.

## Data Availability

The data presented in this study are available on request from the corresponding author.
